# Prevalence of iron overload in patients with chronic kidney disease on peritoneal dialysis: A scoping review

**DOI:** 10.1002/hsr2.2255

**Published:** 2024-09-08

**Authors:** Abdulqadir J. Nashwan, Mohammad T. Abuawwad, Jaber H. Jaradat, Anas Ibraheem, Mohamed A. Yassin, Mohammad J. J. Taha

**Affiliations:** ^1^ Nursing & Midwifery Research Department Hamad Medical Corporation Doha Qatar; ^2^ Department of Public Health, College of Health Sciences, QU Health Qatar University Doha Qatar; ^3^ Clinical Medicine Department, Kasr Alainy Faculty of Medicine Cairo University Cairo Egypt; ^4^ Faculty of Medicine Mutah University Al‐Karak Jordan; ^5^ Haematology Department King's College Hospital London United Kingdom; ^6^ Hematology and Oncology Department, National Center for Cancer Care & Research Hamad Medical Corporation Doha Qatar

**Keywords:** chelating, chronic kidney disease, ferritin, iron overload, peritoneal dialysis

## Abstract

**Background and Aims:**

Chronic kidney disease (CKD) patients undergoing peritoneal dialysis (PD) are susceptible to complications, including iron overload, which can significantly impact their prognosis and overall health. This scoping review aimed to study the prevalence and implications of iron overload in CKD patients undergoing PD.

**Methods:**

A comprehensive search was conducted across five databases, leading to the selection of 18 papers for in‐depth analysis. These studies collectively involved 381 PD patients, 60.3% were males.

**Results:**

No consensus was reached regarding the exact diagnostic cutoff for iron overload. The investigations revealed four main aspects: (1) Seven papers identified various factors contributing to iron overload, emphasizing the role of different iron supplements and magnetic resonance imaging's capability to diagnose iron accumulation in organs; (2) Iron overload in young patients was found to hinder growth; (3) Six studies highlighted the adverse effects of iron overload, with cardiac issues being the most significant; (4) Three studies demonstrated the efficacy of iron‐chelating agents, Deferoxamine and Deferasirox, in treating iron overload patients undergoing PD. Overall, the estimated prevalence of liver iron overload in CKD patients on PD ranges from approximately 10% to 28.6%, which is far lower than the prevalence of 75% elegantly shown in HD patients.

**Conclusion:**

While iron overload was a significant concern for CKD patients undergoing PD in the past, it is less common in the current era due to advancements in treatments, such as erythropoiesis‐stimulating agents. Treatment with specific chelation agents has proven beneficial, but there is also a risk of adverse effects, necessitating meticulous monitoring and timely intervention.

## 1 INTRODUCTION

Peritoneal dialysis (PD) is a kidney replacement treatment modality received by an estimated 11% of chronic kidney disease (CKD) patients on dialysis worldwide.^1^ It is a cost‐effective alternative intended to provide relative ease of use, protect patients from significant blood pressure alterations in patients on hemodialysis (HD), and maintain residual kidney function.^1^, ^2^ Although iron is a vital component and is strictly regulated by the body, iron overload, also known as secondary hemosiderosis, may arise in many conditions, including chronic transfusions. It is associated with multiorgan damage, such as liver, heart, and pancreas damage, due to the lack of enough protective and excretory mechanisms of iron. Iron is crucial in reducing free radical production in the cells.^3^, ^4^

Iron overload associated with chronic transfusion has long been highlighted in the literature. Since patients undergoing dialysis receive erythropoiesis‐stimulating agents (ESAs) to correct adverse anemia, they are often administered iron supplementation to ensure sufficient iron stores.^5^ Patients undergoing HD often receive intravenous (IV) iron, resulting in significant iron accumulation; however, patients undergoing PD often take oral iron supplements because their iron requirements are generally lower.^1^, ^6^ Consequently, iron overload in dialysis patients may severely impair their health. Iron overload in HD patients has been investigated in several studies, and hepatic, cardiac, and multiorgan effects have been studied.^7^, ^8^, ^9^ PD and HD differ significantly in their impact on iron metabolism. PD involves the continuous exchange of dialysate through the peritoneal cavity, which can lead to a more gradual and consistent removal of waste and excess minerals, including iron.^8^ In contrast, HD involves intermittent sessions where blood is filtered through an external machine, which can cause more abrupt shifts in iron levels.^9^ These differences highlight the need for tailored management strategies to address iron overload in patients undergoing PD.

Several approaches have been employed to manage iron overload and prevent its associated complications. These therapies can be broadly categorized as follows: (1) phlebotomy,^10^ (2) Iron chelators,^3^ (3) Dietary Modifications.^11^ Deferoxamine (DFO), the first iron chelator used to treat excess iron, is primarily administered parenterally owing to its limited oral absorption.^3^ Deferasirox (DFX) is an oral iron chelator that offers a convenient once‐daily administration. DFX has shown efficacy in reducing serum ferritin and liver iron content in patients with transfusion‐dependent conditions, such as sickle cell disease and β‐thalassemia.^12^ In patients with CKD, most iron chelators are unsuitable, where DFX offers a promising solution.^13^ However, its high cost, potential side effects, and the need for close monitoring, along with limited comprehensive studies on its efficacy and safety, present notable challenges according to a recent review that examined the potential and difficulties of using DFX in CKD patients undergoing dialysis.^13^

In this scoping review, we aimed to investigate the prevalence of iron overload in CKD patients undergoing PD and emphasize the possible correlations with different iron supplements. The diagnostic methods and criteria for iron overload in patients undergoing PD and consequences and treatment regimens are also discussed.

## 2 METHODS

### 2.1 Rationale

The present review was conducted to synthesize publicly available or published data on iron overload in patients with CKD undergoing PD treatment. This scoping review adheres to the guidelines outlined in the Preferred Reporting Items for Systematic Reviews and Meta‐Analyses extension for Scoping Reviews (PRISMA‐ScR) checklist.^14^

### 2.2 Protocol registration

No specific review protocol was registered for this scoping review.

### 2.3 Eligibility criteria

The inclusion criteria for this review were as follows:

- Published in English.
- No restrictions on the publication date.
- Any study methodology except for reviews, editorials, letters, commentaries, or any secondary articles.

### 2.4 Search strategies

A comprehensive search was conducted from July 15 to 25, 2022, across five electronic databases: PubMed, Scopus, MedRxiv, Cochrane, and Google Scholar. The keywords used for the search are illustrated in Table 1. Medical Subject Headings (MeSH) terms were added when applicable to ensure the retrieval of all relevant studies based on their indexed terms in the included databases.

**Table 1:** Search terms used in databases.

Category	Term
CKD	“Chronic kidney diseases” OR “chronic renal disease” OR “chronic renal failure” OR “Chronic Kidney failure” OR “chronic nephropathy” OR “long‐term kidney disease” OR “end‐stage kidney disease” OR “chronic kidney dysfunction”
AND	
Peritoneal dialysis	“Peritoneal dialysis” OR “continuous cycling peritoneal dialysis” OR “Automated peritoneal dialysis” OR “peritoneal pheresis” OR “peritoneal apheresis”
AND	
Iron overload	“Iron overload” OR “iron accumulation” OR “iron toxicity” OR “hemochromatosis” OR “bronze diabetes” OR “iron‐storage disease” OR “Acquired hemochromatosis”

Abbreviation: CKD, chronic kidney disease.

### 2.5 Data filtration process

Four reviewers (JJ, AI, MT, MA) performed the filtration process through three stages:

- Articles were initially filtered by title.
- The next stage involved filtering by abstract.
- The final stage included a full‐text review.

At each stage, two reviewers independently assessed each article. Discrepancies were resolved by a third author or through discussion.

### 2.6 Data extraction

Data extraction was conducted independently by four reviewers (JJ, AI, MT, MA) using a designated data extraction form created with Google Forms. The form was divided into four main sections:

- General Information: Paper ID, authors, publication year, and location of the study.
- Study Characteristics: Study type, number of patients, study duration, age group (adults or pediatrics), mean age, sex, number of CKD patients, number of patients undergoing PD, and type of PD.
- Clinical Characteristics: Number of patients with iron overload, serum levels of ferritin, iron, transferrin, total iron‐binding capacity (TIBC), transferrin saturation (TSAT), and any relevant clinical findings.
- Study Outcomes: Summary of the study's main findings.

## 3 RESULTS

**Figure 1:**
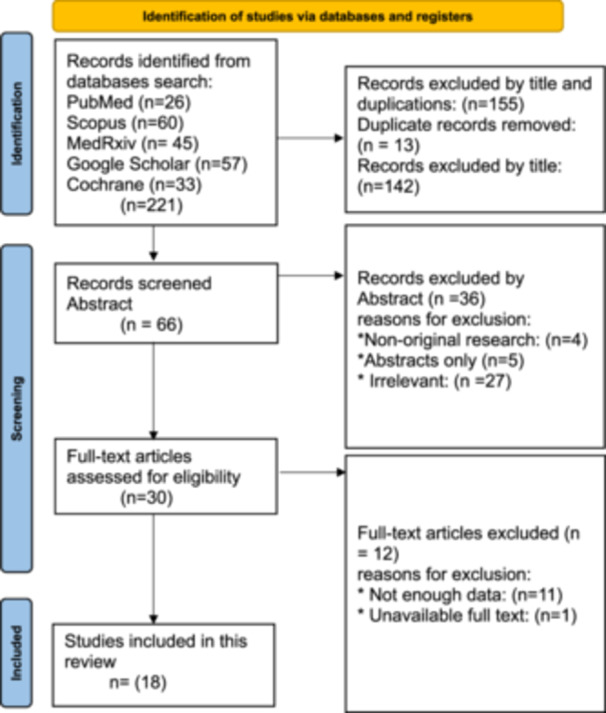
PRISMA‐ScR chart summarizing filtration process.

Our search yielded 221 articles, reduced to 62 records after abstract filtration and 30 after full‐text review. After full text review, 18 papers were included in this review describing 915 patients, of whom 381 were patients undergoing PD (Table 2). Figure 1 includes a PRISMA‐style chart summarizing the filtration process.

**Table 2:** General characteristics of included articles.

Paper ID	Publication year	Country	Methodology	No. of patients	Age group investigated^a^	Mean age	Sex (% males)	No. of PD patients	Iron overload setting^b^
Stanbaugh et al.^15^	1983	USA	Case Report	1	Adults	32	100%	1	N/A
Querfeld et al.^16^	1988	USA	Cross‐sectional (Retrospective)	18	All age groups	15.5	N/A	18	SF > 200 ng/mL
Andreoli & Cohen^17^	1989	USA	Cross‐sectional (Prospective)	3	Juvenile	16.3	66.67%	3	SF > 2000 ng/mL
Ruggian et al.^18^	1995	USA	Case Report	1	Adults	44	100%	1	N/A
Kelly & O'Rourke^19^	2001	USA	Case Report	1	Adults	60	One female	1	N/A
Wu et al.^20^	2004	Taiwan	Cross‐sectional (Prospective)	102	Adults	47.8	36.2%	102	N/A
Ali & Al‐Mashadani^21^	2006	Iraq	Cross‐sectional (Prospective)	20	Adults	49.6	N/A	10	N/A
Saglam et al.^22^	2007	Turkey	Cross‐sectional (Retrospective)	12	Adults	52.5	50%	12	SF > 400 ng/mL
Yusuf et al.^23^	2008	USA	Case Report	1	Adults	43	N/A	1	N/A
Jairam et al.^24^	2010	India	Cross‐sectional (Prospective)	74	Adults	36	72.9%	6	TSAT > 50%
Ruiz‐Jaramillo et al.^25^	2011	Mexico	Cross‐section (prospective) and case‐control	48	Pediatrics	12.55	49%	48	SF > 800 ng/mL
Bavbek et al.^26^	2014	Turkey	Cross‐sectional (Retrospective)	58	Adults	42.1	N/A	58	N/A
Casimiro de Almeida et al.^27^	2015	Guatemala	Cross‐sectional (Prospective)	54	All age groups	13	41%	27	SF > 250 ng/dL
Issad et al.^28^	2017	France	Cross‐sectional (Prospective)	32	Adults	64.5	53.1%	32	Liver iron concentration >50 mmol/g
Hiratsuka et al.^29^	2017	Japan	Cross‐sectional (Retrospective)	132	Adults	61.9	49%	25	SF > 300 ng/dL
Yii et al.^30^	2018	Australia	Case Report	1	Adults	53	100%	1	N/A
Rostoker et al.^31^	2022	France	Cohort (prospective)	7	Adults	61	N/A	7	N/A
Lee et al.^32^	2023	South Korea	Cross‐sectional (Retrospective)	350	Adults	54.7	67.1%	28	SF > 600 ng/mL
**Overall**				**915**		**47.3**	**60.3%**	**381**	

Abbreviation: PD, peritoneal dialysis.

a Age groups: Adults >18 years, pediatric <18 years.

b SF: serum ferritin; TSAT: transferrin saturation.

### 3.1 Characteristics and sources of the included studies

Included articles ranged according to publication year from 1983 to 2023 across several countries and areas worldwide. Most included articles were cross‐sectional studies (*n* = 12), with one cohort study and five case reports. Patients had a mean age of 47.3 years, although two articles described pediatric patients only (<18 years).^17^, ^25^ Generally, among studies that described the sex of their respective populations, 60.3% of patients were males.

Most patients received Continuous Ambulatory Peritoneal Dialysis (CAPD), although some cases included Automated Peritoneal Dialysis (APD). No specific cutoff value for iron overload was agreed upon among the included articles; however, serum ferritin was the most popular diagnostic test.

Four main aspects were discussed in the included articles, and accordingly, the present study summarizes evidence within these sub‐topics:

#### 3.1.1 Incidence and diagnosis of iron overload patients on patients undergoing PD

Of the included articles, seven studies discussed detecting iron overload in PD patients within certain circumstances or in comparison with other clinical settings. Of these articles, Ali and Mashadani^21^ described 10 PD patients receiving recombinant human erythropoietin (rHuEpo) and iron dextran after dialysis without significant elevation in iron markers. In a similar context, Saglam et al.^22^ investigated the impact of IV iron‐sucrose on oxidative stress in CAPD patients, monitoring iron accumulation and clearance. Saglam et al.^22^ found that iron stores may influence oxidative stress before administration, suggesting that if baseline ferritin was optimal, IV iron‐sucrose is safe to administer in CAPD patients.

An interesting comparison was conducted by Hiratsuka et al.^29^ in which the authors compared the impact of ferric citrate hydrate (FCH), an iron‐based phosphate binder, on iron accumulation with HD and PD. None of their HD patients suffered from iron overload. In contrast, five patients undergoing PD developed excess iron, which suggested that the dialysis type influenced FCH and its role in iron overload incidence.^29^

Casimiro de Almeida et al.^27^ assessed the impact of dialysis modalities (HD and PD) in pediatrics with end‐stage kidney disease (ESKD) on growth and inflammation. Two main markers significantly correlated to growth restriction and inflammatory status: serum ferritin and Hepcidin‐25. HD patients reported significantly higher iron accumulation compared to patients undergoing PD. Of PD patients, 55% were stunted in growth, 85% were anemic, and Hepcidin‐25 showed marked twofold or less elevation, indicating significant inflammation, although less than patients on HD.^27^

Three articles utilized magnetic resonance imaging (MRI) to assess iron metabolism and accumulation in patients undergoing PD, especially in the liver and spleen. Querfeld et al.^16^ and Issad et al.^28^ aimed to assess iron overload in pediatric and adult patients using MRI imaging of the liver and spleen. The signal intensity observed in the livers and spleens of patients undergoing PD was descriptive of iron accumulation, proving MRI effective in diagnosing iron overload. Similarly, Rostoker et al.^31^ conducted a cohort study over 6 years, using MRI to explore liver tropism for iron sucrose, ferric carboxymaltose, and iron isomaltose in seven patients with CKD and iron overload on PD. Iron sucrose was correlated with higher levels in the liver and spleen, affecting liver structure, although the aforementioned article was not devoted specifically to patients undergoing HD or PD but rather to an in‐vivo pharmacokinetic analysis.^31^ Therefore, the estimated prevalence of liver iron overload in CKD patients on PD ranges from approximately 10% to 28.6%, which is far lower than the prevalence of 75% elegantly shown in HD patients.^7^ However, these proportions need to be validated through a systematic review with a larger sample size to ensure more accurate and reliable estimates.

#### 3.1.2 Consequences of iron overload in patients undergoing PD

Six studies investigated the adverse effects of iron excess.^15^, ^20^, ^24^, ^25^, ^26^, ^32^ All studies concluded that iron overload is one of the reasons for sudden mortality in patients undergoing PD. These incidences of sudden death in patients undergoing PD were usually due to cardiac reasons. According to Bavbek et al.,^26^ CAPD patients are in danger of significant arrhythmia and abrupt cardiac death from the repeated infusion of iron or blood. Lee et al.^32^ also found that a high ferritin level was related to an increased risk of all‐cause death in patients undergoing PD and that this impact was independent of age, anemia, and volume status. According to Ruiz‐Jaramillo et al.,^25^ three or more cardiovascular risk factors were exhibited twice in iron excess patients, and iron overload patients had a higher proportion of malnutrition, hypertension, and hemoglobin than patients without iron overload. In two papers, namely, Jairam et al.^24^ and Ruiz‐Jaramillo et al.,^25^ inflammatory markers such as CRP, IL‐6, and tumor necrosis factor‐a were also notably elevated in iron overload patients.^24^, ^25^ However, according to,^24^ the activation of these inflammatory markers was more evident in adult patients who received IV iron, while^25^ observed similar findings in infants and adolescents. Wu et al.^20^ investigated QT depression in 102 patients undergoing PD by monitoring their electrocardiogram changes in correlation with the changes in iron stores. They concluded that a value of TSAT less than 35% was a safe iron status in patients undergoing PD, considering the risk of arrhythmias.

#### 3.1.3 Treatment for PD‐related iron overload

Three of the articles were concerned with treatment modalities to manage iron overload in patients undergoing PD. Andreoli et al.^17^ used intraperitoneal Deferoxamine (DFO) therapy in three pediatric patients on CAPD, which showed considerable iron removal and negative iron balance in patients originally suffering from iron overload. Nevertheless, despite DFO being knowingly capable of reducing serum ferritin levels and hepatic iron content, it did not reduce liver iron content but did reduce serum iron levels.^17^

Two papers used Deferasirox (DFX) as an Iron chelating agent. Yii et al.^30^ reported the case of a 53‐year‐old patient with beta‐thalassemia major and CKD on CAPD. They used DFX at a dose of 1000 mg/day, which showed moderate efficacy in reducing iron overload. Yusuf et al.^23^ reported the first case of recurrent DFX‐related symptomatic hypocalcemia in a 43‐year‐old patient undergoing PD treated with DFX. The patient was anemic and received long‐term blood transfusions and erythropoietin treatment.^23^

#### 3.1.4 Relation to porphyria cutanea tarda

The present study included two case reports of PCT in ESKD patients receiving PD. Kelly and O'Rourk^19^ discussed the available therapeutic options for treating a 60‐year‐old white woman with ESKD on PD complicated by sporadic PCT. Plasma levels of fractionated porphyrins were high, uroporphyrin 53.4 mg/dL, heptacarboxyporphyrin 47.8 mg/dL, hexacarboxyporphyrin 10.8 mg/dL, pentacarboxyporphyrin 2.9 mg/dL, coproporphyrin 1.6 mg/dL. Therefore, estrogen replacement therapy, furosemide, and polysaccharide‐iron complex supplements were all stopped to minimize the flair‐up. Due to ongoing anemia, PCT was treated with more than 4 months of bi‐weekly hydroxychloroquine and sun avoidance instead of phlebotomy. However, her skin lesions continued to worsen. “In our patient, phlebotomy may be necessary to achieve improvement,” the authors wrote. This happened in complete remission after undergoing monthly phlebotomy for 20 weeks with steady hemoglobin levels.^19^

Ruggian et al.^18^ reported a 44‐year‐old epileptic white man with ESKD on CAPD who PCT complicated due to the offending drug phenytoin. Although a 24‐h urine collection showed normal concentrations of fractionated porphyrins, his plasma levels were high for heptacarboxyporphyrins, uroporphyrins, and hexacarboxyporphyrins. After 4 months of frequent phlebotomies and switching the phenytoin to carbamazepine, the skin lesions had completely resolved.^18^

## 4 DISCUSSION

Iron overload is a known complication in the treatment of CKD patients undergoing PD, often necessitating additional management and control. This scoping review examined adverse incidences of iron overload in CKD patients undergoing PD. Our findings indicate that 60.3% of the patients were male. The male gender is associated with higher serum ferritin concentrations and elevated hepcidin‐25 levels.^33^ Despite CKD being more prevalent among women, they are less likely to commence dialysis. This discrepancy may be attributed to the misinterpretation of women's glomerular filtration rate (GFR), slower progression to ESKD, and various socioeconomic factors.^34^

The most important finding of this study is that no specific level of serum ferritin or any other iron marker was agreed on in the included articles as the clear determinant of iron overload. This finding goes shoulder‐to‐shoulder with the evidence of why the MRI has become the gold‐standard method for diagnosing and monitoring iron overload instead of serum ferritin.^35^ The latter marker of different levels was adapted as criteria in a few studies.^16^, ^17^, ^22^, ^25^, ^27^, ^29^, ^32^ At the same time, TSAT was adopted by Jairam et al.,^24^ and liver iron concentration was used in Issad et al.'s^28^ study. Diagnosing hemochromatosis or iron excess can be confusing, but values like serum ferritin, TSAT, and liver iron concentration are all used in clinical practice. Some consider serum ferritin higher than 300 ng/mL in males and higher than 200 ng/m in menstruating females. Still, these values are prone to errors as serum ferritin is an acute phase reactant, so it can be elevated in inflammations or infections.^36^ These levels were adapted from papers published in 1998, which begs whether these levels are clinically accurate and generalizable.^37^, ^38^

One interesting aspect was addressed in Casimiro de Almeida et al.'s^27^ study, where the authors addressed the alteration in growth due to iron imbalance. It is known that iron deficiency results in growth restriction.^39^ Still, Casimiro de Almeida et al.^27^ highlight the impact of iron excess and inflammatory state on growth restriction, a described phenomenon, often in the context of iron overload in beta‐thalassemia patients.^40^, ^41^

Different types of iron supplementation were used in the included studies, each with a different rate of iron overload incidence and a different adverse reaction profile. rHuEpo and iron dextran have been used^21^; rHuEpo is an older drug, which has been recorded to turn iron deficiency into iron overload if improperly used, especially in resistant cases requiring IV iron administration.^42^ On the other hand, iron sucrose has been used in Saglam et al.^22^ and Rostoker et al.^31^ Both studies indicated iron overload due to iron‐sucrose administration to patients undergoing PD and tried to develop preadministration criteria for patient evaluation. However, according to Rostoker et al.,^31^ ferric carboxymaltose, and iron isomaltose were safer and less prone to accumulation in the liver, which is supported in the literature by clinical trials comparing the administration of both iron supplements.^43^, ^44^

ESKD patients undergoing PD are at risk of severe morbidity up to mortality due to iron overload. This review found that iron overload can cause death by inducing fatal cardiac arrhythmias.^15^, ^20^, ^24^, ^25^, ^26^, ^32^ Iron overload could lead to cardiomyopathy, inducing preventable heart failure, starting as a diastolic dysfunction and arrhythmias.^45^ Management of iron overload in CKD patients on PD involves a combination of continuous monitoring (e.g., MRI) and iron chelation.^46^, ^47^ In our cohort, two cases were presented where iron chelating agents DFO and DFX were administered. DFO, a common iron‐chelating agent, reduced serum iron effectively but was less effective in reducing liver iron.^17^ This had been investigated in Drakonaki et al.^48^ study on patients suffering from beta‐thalassemia. They found that liver, spleen, and bone marrow iron content was less effectively reduced with DFO chelation than combined therapy. In their group of 21 patients, 38% showed decreased liver iron, 33.3% showed decreased spleen iron, and 33.3% showed bone marrow iron reduction.^48^ DFX demonstrated lower efficacy in lowering serum ferritin in the case presented by Yii et al.^30^ It was linked to adverse hypocalcemia in the case presented by Yusuf et al.^23^ In the literature, DFX was associated with worse serum ferritin reduction and more prevalent adverse events, including a decline in renal tubular function (i.e., eGFR).^49^ Cassinerio et al.^50^ proposed a combination of DFO and DFX, suggesting that it would improve iron chelation in resistant cases and reduce the incidence of adverse events. The study also found that the combination can assist in reducing liver iron concentration and protecting against iron‐related cardiac manifestations.

Two articles described PCT in their patients.^18^, ^19^ Various susceptibility factors render patients prone to PCT, including genetic factors, environmental and socioeconomic aspects, and relation to some antiepileptic drugs.^18^, ^51^ Both cases were managed through phlebotomies, within the standard PCT treatment. The first‐line treatment for PCT in patients with normal kidney function is phlebotomy with removal of 450 mL every couple of weeks and low dose hydroxychloroquine along with interrupting the triggers and protecting the skin from sunlight until porphyrin levels return to normal.^52^, ^53^

Limitations to this review include the scarcity of evidence, as research papers directed towards iron overload in CKD patients undergoing PD are rather rare. Thus, heterogeneity in the included articles in this review was inevitable, and the conclusions made in the present review require further investigation and reassessment. Additionally, due to the extreme heterogeneity of the articles, we transitioned from an initially planned systematic review to a scoping review. Our focus on English‐language studies, due to resource constraints, may have limited the inclusion of relevant literature in other languages. Furthermore, while we applied a standardized data extraction method to minimize potential bias, some variability may still be present. Nevertheless, continuous monitoring of serum ferritin in patients undergoing PD is important to protect them against iron overload and prevent serious adverse events that could develop otherwise. An additional limitation of this review is the inclusion of studies published before 1995. During this period, the treatment landscape for CKD patients, particularly those undergoing PD, differed significantly from the current era. The widespread use of IV iron (dextrans) and blood transfusions was more prevalent before the introduction and common use of ESAs. Consequently, the findings from these older studies may not accurately reflect the current clinical practices and management strategies for iron overload in PD patients. To address this limitation, future reviews should consider excluding studies conducted before the widespread adoption of ESAs to ensure that the synthesized data accurately represent contemporary treatment modalities and outcomes.

Overall, the prevalence of iron overload and its consequences in the present review can contradict the results published by Rostoker et al.^54^ in 2019 since most of the included articles did not rely on a solid background for diagnosing iron overload, in particular quantitative MRI. Therefore, the conclusions made in the present review require further investigation and reassessment. Unlike HD, patients undergoing PD in the ESA era have a lower need for iron store replenishment; therefore, iron overload is less common and mostly mild in those patients.^54^ This can be attributed to the fact that iron metabolism differs markedly between HD and PD.^1^ In fact, those rationalizations are the cornerstone for the Kidney Disease Improving Global Outcomes (KDIGO) recommendations and the European Renal Best Practice (ERBP) position statement of more conservative strategies advocated for patients undergoing PD compared to HD, including serum ferritin target of >100 µg/L and oral iron instead of IV therapy; however, the latter route is still reserved as an ESA‐sparing agent for PD patients, who either poorly tolerate/respond to oral iron or having high iron requirements.^5^, ^54^, ^55^

That said, patients undergoing PD nowadays are considered to have less risk of iron overload together with the current use of ferric carboxymaltose and iron isomaltoside which have been shown minimal accumulation in liver and spleen in comparison to iron sucrose in patients undergoing PD.^31^ This fact explains the failure to reach a consensus closure to this review since more than two‐thirds of patients undergoing PD in the included articles are from middle and low‐income countries. Consequently, large use of IV iron (mainly iron sucrose and iron gluconate due to their low prices) as a sparing agent of costly ESA and still large use of blood transfusions can lead to an epidemic of iatrogenic iron accumulation in patients undergoing PD which requires specific treatment to mitigate potentially serious consequences. In addition, ferric citrate has to be carefully monitored because it can induce a substantial proportion of patients with iron overload.^5^

## 5 CONCLUSION

Iron accumulation following PD in CKD patients remains a mild‐moderate risk that necessitates monitoring and management, even though its incidence is less common today due to current guidelines for diagnosing and treating such patients. The estimated prevalence of iron overload in CKD patients undergoing PD ranges from approximately 10% to 28.6%; considering the limitations of the included studies. The use of iron chelators like DFO and DFX helps prevent iron overload. Future research is needed to refine our understanding of this condition, enhance management strategies, and ensure optimal patient outcomes. These efforts will help further mitigate the risks and improve the clinical care of CKD patients undergoing PD.

## AUTHOR CONTRIBUTIONS

**Abdulqadir J. Nashwan, Mohammad T. Abuawwad, and Mohamed A. Yassin**: Conceptualization. **Abdulqadir J. Nashwan, Mohammad T. Abuawwad, Jaber H. Jaradat, Anas Ibraheem, Mohamed A. Yassin, and Mohammad J. J. Taha**: Literature search, writing‐ original draft, editing, review‐ final draft. All authors read and approved the final manuscript.

## CONFLICT OF INTEREST STATEMENT

Abdulqadir J. Nashwan is an Editorial Board member of Health Science Reports and co‐author of this article. He is excluded from editorial decision‐making related to the acceptance of this article for publication in the journal. The other authors declare no conflicts of interest.

## ETHICS STATEMENT

Ethical approval was not required for this scoping review. Scoping reviews synthesize publicly available or published data and do not involve the collection of primary data from human or animal subjects.

## TRANSPARENCY STATEMENT

The lead author Abdulqadir J. Nashwan affirms that this manuscript is an honest, accurate, and transparent account of the study being reported; that no important aspects of the study have been omitted; and that any discrepancies from the study as planned (and, if relevant, registered) have been explained.

## Data Availability

The data that support the findings of this study are openly available in figshare at https://doi.org/10.6084/m9.figshare.24265561.v1. The data [Dataset] supporting the findings of this study are openly available in the repository [figshare] at [https://doi.org/10.6084/m9.figshare.24265561.v1].

## References

[1] Auguste BL , Bargman JM . Peritoneal dialysis prescription and adequacy in clinical practice: core curriculum 2023. Am J Kidney Dis. 2023;81(1):100‐109. 36208963 10.1053/j.ajkd.2022.07.004

[2] Mehrotra R , Devuyst O , Davies SJ , Johnson DW . The current state of peritoneal dialysis. J Am Soc Nephrol. 2016;27(11):3238‐3252. 27339663 10.1681/ASN.2016010112 PMC5084899

[3] Entezari S , Haghi SM , Norouzkhani N , et al. Iron chelators in treatment of iron overload. J Toxicol. 2022;2022:4911205. 35571382 10.1155/2022/4911205 PMC9098311

[4] Kohgo Y , Ikuta K , Ohtake T , Torimoto Y , Kato J . Body iron metabolism and pathophysiology of iron overload. Int J Hematol. 2008;88(1):7‐15. 18594779 10.1007/s12185-008-0120-5 PMC2516548

[5] Rostoker G , Vaziri ND , Fishbane S . Iatrogenic iron overload in dialysis patients at the beginning of the 21st century. Drugs. 2016;76(7):741‐757. 27091216 10.1007/s40265-016-0569-0 PMC4848337

[6] Nashwan AJ , Yassin MA , Mohamed Ibrahim MI , Abdul Rahim HF , Shraim M . Iron overload in chronic kidney disease: less ferritin, more T2(*)MRI. Front Med. 2022;9:865669. 10.3389/fmed.2022.865669 PMC8977522 35386917

[7] Nashwan AJ , Yassin MA , Abd‐Alrazaq A , et al. Hepatic and cardiac iron overload quantified by magnetic resonance imaging in patients on hemodialysis: a systematic review and meta‐analysis. Hemodial Int. 2023;27(1):3‐11. 36397717 10.1111/hdi.13054

[8] Rostoker G , Vaziri ND . Iatrogenic iron overload and its potential consequences in patients on hemodialysis. Presse Med. 2017;46(12 Pt 2):e312‐e328. 29153377 10.1016/j.lpm.2017.10.014

[9] Nashwan AJ , Yassin MA , Abd‐Alrazaq A , Shuweihdi F , Abdul Rahim HF , Shraim M . The prevalence of cardiac and hepatic iron overload in patients with kidney failure: a protocol for systematic review and meta‐analysis. Health Sci Rep. 2022;5(4):e692. 35702513 10.1002/hsr2.692 PMC9178349

[10] Porter JB , de Witte T , Cappellini MD , Gattermann N . New insights into transfusion‐related iron toxicity: implications for the oncologist. Crit Rev Oncol Hematol. 2016;99:261‐271. 26806144 10.1016/j.critrevonc.2015.11.017

[11] Milman NT . Managing genetic hemochromatosis: an overview of dietary measures, which may reduce intestinal iron absorption in persons with iron overload. Gastroenterol Res. 2021;14(2):66‐80. 10.14740/gr1366 PMC8110241 34007348

[12] Shah N . Advances in iron chelation therapy: transitioning to a new oral formulation. Drugs Context. 2017;6:1‐10. 10.7573/dic.212502 PMC5499896 28706555

[13] Nashwan A , Yassin M . Deferasirox in patients with chronic kidney disease: assessing the potential benefits and challenges. J Blood Med. 2023;14:589‐594. 38047247 10.2147/JBM.S415604 PMC10693276

[14] Tricco AC , Lillie E , Zarin W , et al, PRISMA Extension for Scoping Reviews (PRISMA‐ScR) . PRISMA extension for scoping reviews (PRISMA‐ScR): checklist and explanation. Ann Intern Med. 2018;169(7):467‐473. 30178033 10.7326/M18-0850

[15] Stanbaugh GH , Gillit AHD , Reichel GW , Stranz M . Iron chelation therapy in CAPD: a new and effective treatment of iron overload disease in esrd patients. Perit Dial Int. 1983;3(2):99‐101.

[16] Querfeld U , Dietrich R , Taira RK , Kangarloo H , Salusky IB , Fine RN . Magnetic resonance imaging of iron overload in children treated with peritoneal dialysis. Nephron. 1988;50(3):220‐224. 3226457 10.1159/000185162

[17] Andreoli SP , Cohen M . Intraperitoneal deferoxamine therapy for iron overload in children undergoing CAPD. Kidney Int. 1989;35(6):1330‐1335. 2770113 10.1038/ki.1989.131

[18] Ruggian JC , Fishbane S , Demento FJ , Maesaka JK , Frei GL . Porphyria cutanea tarda in a patient on chronic ambulatory peritoneal dialysis. J Am Soc Nephrol. 1996;7(3):397‐402. 8704104 10.1681/ASN.V73397

[19] Kelly MA , O'Rourke KD . Treatment of porphyria cutanea tarda with phlebotomy in a patient on peritoneal dialysis. J Am Acad Dermatol. 2001;44(2 suppl):336‐338. 11174409 10.1067/mjd.2001.102668

[20] Wu V‐C , Huang J‐W , Wu M‐S , et al. The effect of iron stores on corrected QT dispersion in patients undergoing peritoneal dialysis. Am J Kidney Dis. 2004;44(4):720‐728. 15384024

[21] Ali B , Al‐Mashadani Z . Comparison of serum iron and total iron binding capacity between hemo and peritoneal dialysis patients. Ibn al‐Haitham J Pure Appl Sci. 2017;19(1):49‐55.

[22] Saglam F , Cavdar C , Uysal S , Cavdar Z , Camsari T . Effect of intravenous iron sucrose on oxidative stress in peritoneal dialysis patients. Ren Fail. 2007;29(7):849‐854. 17994454 10.1080/08860220701573566

[23] Yusuf B , McPhedran P , Brewster UC . Hypocalcemia in a dialysis patient treated with deferasirox for iron overload. Am J Kidney Dis. 2008;52(3):587‐590. 18534729 10.1053/j.ajkd.2008.03.034

[24] Jairam A , Das R , Aggarwal P , et al. Iron status, inflammation and hepcidin in ESRD patients: the confounding role of intravenous iron therapy. Indian J Nephrol. 2010;20(3):125‐131. 21072151 10.4103/0971-4065.70840 PMC2966977

[25] Ruiz‐Jaramillo MC , Guizar‐Mendoza JM , Amador‐Licona N , et al. Iron overload as cardiovascular risk factor in children and adolescents with renal disease. Nephrol Dial Transplant. 2011;26(10):3268‐3273. 21372265 10.1093/ndt/gfr044

[26] Bavbek N , Yilmaz H , Erdemli HK , Selcuki Y , Duranay M , Akçay A . Correlation between iron stores and QTc dispersion in chronic ambulatory peritoneal dialysis patients. Ren Fail. 2014;36(2):187‐190. 24059284 10.3109/0886022X.2013.836750

[27] Casimiro de Almeida J , Lou‐Meda R , Olbert M , et al. The growth attainment, hematological, iron status and inflammatory profile of Guatemalan juvenile end‐stage renal disease patients. PLoS One. 2015;10(10):e0140062. 26445018 10.1371/journal.pone.0140062 PMC4596869

[28] Issad B , Ghali N , Beaudreuil S , Griuncelli M , Cohen Y , Rostoker G . Hepatic iron load at magnetic resonance imaging is normal in most patients receiving peritoneal dialysis. Kidney Int Rep. 2017;2(6):1219‐1222. 29270530 10.1016/j.ekir.2017.07.005 PMC5733676

[29] Hiratsuka M , Koyama K , Sengo K , et al. Long‐term iron accumulation in dialysis patients treated with ferric citrate hydrate: a single‐center, 80‐week retrospective study in Japan. Ren Replace Ther. 2017;3(1):37.

[30] Yii E , Doery JC , Kaplan Z , Kerr PG . Use of deferasirox (Exjade) for iron overload in peritoneal dialysis patients. Nephrology. 2018;23(9):887‐889. 29663590 10.1111/nep.13389

[31] Rostoker G , Lepeytre F , Merzoug M , et al. Differential pharmacokinetics of liver tropism for iron sucrose, ferric carboxymaltose, and iron isomaltoside: a clue to their safety for dialysis patients. Pharmaceutics. 2022;14(7):1408. 35890303 10.3390/pharmaceutics14071408 PMC9323124

[32] Lee HS , Noh HM , An JN , Song YR , Kim SG , Kim JK . Elevated ferritin levels associated with high body fat mass affect mortality in peritoneal dialysis patients. Nutrients. 2023;15(9):2149. 37432308 10.3390/nu15092149 PMC10180848

[33] Gromadzka G , Wierzbicka D , Litwin T , Przybyłkowski A . Difference in iron metabolism may partly explain sex‐related variability in the manifestation of Wilson's disease. J Trace Elem Med Biol. 2020;62:126637. 32937238 10.1016/j.jtemb.2020.126637

[34] Cobo G , Hecking M , Port FK , et al. Sex and gender differences in chronic kidney disease: progression to end‐stage renal disease and haemodialysis. Clin Sci. 2016;130(14):1147‐1163. 10.1042/CS20160047 27252402

[35] Paisant A , d'Assignies G , Bannier E , Bardou‐Jacquet E , Gandon Y . MRI for the measurement of liver iron content, and for the diagnosis and follow‐up of iron overload disorders. Presse Med. 2017;46(12):e279‐e287. 29133084 10.1016/j.lpm.2017.10.008

[36] McDowell LA , Kudaravalli P , Sticco KL . StatPearls Publishing Copyright ©. Iron Overload. StatPearls. 2023. FL: StatPearls Publishing LLC; 2023. 30252387

[37] Powell LW . Diagnosis of hemochromatosis. Ann Intern Med. 1998;129(11):925‐931. 9867744 10.7326/0003-4819-129-11_part_2-199812011-00002

[38] Cogswell ME . Iron overload, public health, and genetics: evaluating the evidence for hemochromatosis screening. Ann Intern Med. 1998;129(11):971‐979. 9867750 10.7326/0003-4819-129-11_part_2-199812011-00008

[39] De Andrade Cairo RC , Rodrigues Silva L , Carneiro Bustani N , Ferreira Marques CD . Iron deficiency anemia in adolescents; a literature review. Nutr Hosp. 2014;29(6):1240‐1249. 24972460 10.3305/nh.2014.29.6.7245

[40] Langer AL Beta‐Thalassemia. In: Adam MP , Mirzaa GM , Pagon RA , et al.editors., eds. GeneReviews(®). Seattle (WA): University of Washington, Seattle Copyright © 1993‐2023, University of Washington, Seattle. GeneReviews is a registered trademark of the University of Washington, Seattle. All rights reserved 1993.

[41] Galanello R , Origa R . Beta‐thalassemia. Orphanet J Rare Dis. 2010;5:11. 20492708 10.1186/1750-1172-5-11 PMC2893117

[42] Tarng DC , Huang TP , Chen TW , Yang WC . Erythropoietin hyporesponsiveness: from iron deficiency to iron overload. Kidney Int. 1999;55:S107‐S118. 10084294

[43] Jose A , Mahey R , Sharma JB , et al. Comparison of ferric carboxymaltose and iron sucrose complex for treatment of iron deficiency anemia in pregnancy‐ randomised controlled trial. BMC Pregnancy Childbirth. 2019;19(1):54. 30717690 10.1186/s12884-019-2200-3 PMC6360702

[44] Christoph P , Schuller C , Studer H , Irion O , De Tejada BM , Surbek D . Intravenous iron treatment in pregnancy: comparison of high‐dose ferric carboxymaltose vs. iron sucrose. J Perinat Med. 2012;40(5):469‐474. 22945271 10.1515/jpm-2011-0231

[45] Gulati V , Harikrishnan P , Palaniswamy C , Aronow WS , Jain D , Frishman WH . Cardiac involvement in hemochromatosis. Cardiol Rev. 2014;22(2):56‐68. 24503941 10.1097/CRD.0b013e3182a67805

[46] Winchester JF . Management of iron overload in dialysis patients. Sem Nephrol. 1986;6(4 suppl 1):22‐26. 3299589

[47] Nashwan AJ , Alkhawaldeh IM , Shaheen N , et al Using artificial intelligence to improve body iron quantification: a scoping review. Blood Rev. 2023;101133. 37748945 10.1016/j.blre.2023.101133

[48] Drakonaki EE , Maris TG , Maragaki S , Klironomos V , Papadakis A , Karantanas AH . Deferoxamine versus combined therapy for chelating liver, spleen and bone marrow iron in β‐Thalassemic patients: a quantitative magnetic resonance imaging study. Hemoglobin. 2010;34(1):95‐106. 20113293 10.3109/03630260903546445

[49] Tanous O , Azulay Y , Halevy R , et al. Renal function in β‐thalassemia major patients treated with two different iron‐chelation regimes. BMC Nephrol. 2021;22(1):418. 34930156 10.1186/s12882-021-02630-5 PMC8691002

[50] Cassinerio E , Orofino N , Roghi A , et al. Combination of deferasirox and deferoxamine in clinical practice: an alternative scheme of chelation in thalassemia major patients. Blood Cells Mol Dis. 2014;53(3):164‐167. 24846580 10.1016/j.bcmd.2014.04.006

[51] Singal AK . Porphyria cutanea tarda: recent update. Mol Gen Metab. 2019;128(3):271‐281. 10.1016/j.ymgme.2019.01.004 30683557

[52] Shah A , Bhatt H . Cutanea Tarda Porphyria. StatPearls. Treasure Island (FL): StatPearls Publishing Copyright © 2023. StatPearls Publishing LLC; 2023.

[53] Salameh H , Sarairah H , Rizwan M , Kuo YF , Anderson KE , Singal AK . Relapse of porphyria cutanea tarda after treatment with phlebotomy or 4‐aminoquinoline antimalarials: a meta‐analysis. Br J Dermatol. 2018;179(6):1351‐1357. 29750336 10.1111/bjd.16741 PMC6230514

[54] Rostoker G , Griuncelli M , Ghali N , Beaudreuil S , Cohen Y , Issad B . Hepatic iron load differs strikingly between peritoneal dialysis and hemodialysis patients. Bulletin de la Dialyse à Domicile. 2019;2(4):181‐191.

[55] Macdougall IC , Bircher AJ , Eckardt KU , et al. Iron management in chronic kidney disease: conclusions from a “kidney disease: improving global outcomes” (KDIGO) controversies conference. Kidney Int. 2016;89(1):28‐39. 26759045 10.1016/j.kint.2015.10.002

